# Economic modeling of polygenic risk prediction of coronary artery disease in childhood

**DOI:** 10.1038/s44325-026-00110-z

**Published:** 2026-03-23

**Authors:** Fouad Bitar, Rana Zareef, Hussain Ismaeel, Roukoz Abou-Karam, Mariam Arabi, Ziad Bulbul, Fadi F. Bitar, Akl C. Fahed

**Affiliations:** 1 Cardiovascular Disease Initiative, Broad Institute of MIT and Harvard, Cambridge, MA USA; 2 Division of Cardiology, Massachusetts General Hospital, Harvard Medical School, Boston, MA USA; 3 Children’s Heart Center, Department of Pediatrics and Adolescent Medicine, American University of Beirut-Medical Center, Beirut, Lebanon; 4 Division of Cardiology, Aman Hospital, Doha, Qatar

**Keywords:** Cardiology, Diseases

## Abstract

Risk factors and subclinical pathophysiology of coronary artery disease (CAD) begin in childhood, yet identifying candidates for primordial prevention remains challenging. Polygenic risk scores (PRS) provide a DNA-based risk marker from birth that can stratify children by lifetime CAD risk. We evaluated the potential clinical utility and cost-effectiveness of PRS-guided CAD prevention in children using health economic modeling. A Markov model compared PRS-guided prevention with standard prevention in a hypothetical group of 10,000 children. Children in the top 20% of PRS (*n* = 2000) were assumed to receive behavioral interventions. Assuming a 10-year CAD incidence of 12% during adulthood among those in the top 20% of the PRS distribution and a conservative 30% relative risk reduction with preventive intervention, PRS-guided prevention was projected to prevent 72 CAD events among 2000 high-risk children (3.6% absolute event reduction), yielding a return on investment of 3614%. PRS enables early, targeted prevention, improving outcomes, and lowering lifetime costs.

## Introduction

Coronary artery disease (CAD) remains the leading cause of morbidity and mortality worldwide. Evidence from longitudinal studies such as the Bogalusa Heart Study and the Young Finns Study shows that atherosclerosis originates in childhood, with early-life risk factors—including obesity, dyslipidemia, and hypertension—tracking into adulthood and predicting both subclinical and clinical atherosclerosis^1,2^. These findings underscore the importance of initiating primordial prevention in childhood to mitigate the lifetime burden of CAD.

Current pediatric screening—primarily relying on lipid profiles and family history—identifies only a fraction of high-risk individuals and often overlooks those with significant genetic predisposition. Polygenic risk scores (PRS) offer a promising solution by aggregating the effects of numerous common genetic variants into a single, quantifiable measure of inherited risk for CAD. Unlike traditional risk factors, PRS provides a lifelong, stable, and independent predictor of risk, uninfluenced by age or lifestyle, enabling early stratification well before clinical disease emerges^3,4^. Although extensively validated in adults, PRS remains largely underutilized in pediatrics, despite its potential for early identification, which could enable targeted interventions during critical windows of vascular development.

Emerging data support the relevance of PRS in youth. High PRS is associated with greater plaque burden and subclinical atherosclerosis, even among children with normal lipid levels^5–7^. Pediatric interventions, such as those implemented in the STRIP trial, have demonstrated durable cardiometabolic benefits when initiated early in life^8^. Furthermore, modeling studies suggest that PRS-guided prevention could reduce lifetime CAD risk by up to 30%^9,10^. However, few studies have evaluated the integration of PRS into pediatric care, and none have assessed its long-term clinical or economic impact, with a gap in cost-effectiveness evidence for pediatric PRS.

In this study, we evaluate the potential of PRS to inform early CAD prevention in children. We first conducted a bibliometric analysis to characterize global research trends and identify gaps in pediatric PRS studies. We then developed a theoretical simulation-based health economic model to estimate the long-term clinical outcomes and cost-effectiveness of implementing PRS-guided CAD prevention strategies in children across high-income settings. To address the scarcity of pediatric CAD-PRS data, we integrated a bibliometric review with a theoretical economic model to project the long-term impact of PRS-guided prevention in childhood.

## Results

### Bibliometric results

Our bibliometric analysis identified 1355 pediatric PRS studies (1999–2024), with 461 focusing on cardiometabolic traits. Strikingly, only three CAD-specific studies emerged in European ancestry cohorts using legacy cohorts (PDAY, Bogalusa, Young Finns)^6,7,11^. This disparity persists despite evidence from adults showing that PRS improves CAD prediction. A companion adult search found 1034 CAD-PRS studies, revealing a 345:1 pediatric CAD research deficit. Publication trends (Supplementary Fig. 1) reveal an accelerating output since 2011 (+3250%), yet CAD constitutes less than 1% of pediatric cardiometabolic PRS research. Geographic analysis revealed that 92% of studies originated from high-income countries, with fewer than 5% involving non-European populations—a critical limitation, given the multi-ancestry evidence of variable PRS performance^12–16^.

Using PDAY data, Salfati et al.^6^ demonstrated that a CAD genetic risk score was significantly associated with early coronary atherosclerosis, but not aortic atherosclerosis. Similarly, Guarischi-Sousa et al.^7^ showed that higher PRS for CAD and LDL cholesterol correlated with a greater early atherosclerotic burden, independent of blood pressure. In contrast, Hernesniemi et al.^11^ found no association between CAD genetic risk scores and subclinical carotid atherosclerosis across three large cohorts.

The three available pediatric CAD polygenic or genetic risk score studies focus on subclinical atherosclerotic phenotypes rather than longitudinal clinical outcomes (myocardial infarction, stroke, or cardiovascular mortality) and therefore do not provide the event rates, transition probabilities, or intervention effect estimates required for a long-term state-transition model. Importantly, none of these studies report intervention effects. In addition, heterogeneity in study design and PRS/GRS construction precludes harmonized derivation of model inputs. Accordingly, adult cohort data were used to inform clinical event transitions and treatment effects, while pediatric studies were used to support the biological relevance of PRS to early atherosclerotic processes preceding clinical coronary artery disease.

This marked evidence gap, particularly the scarcity of pediatric CAD-PRS studies, underscores the need for a theoretical modeling framework to estimate potential long-term clinical and economic impact.

### Clinical scenarios and QALYs

Using the prespecified model inputs, as defined in the methods, the simulation projected that 72 CAD cases could be prevented over 10 years among 2000 high-risk children (top 20% PRS). All model assumptions and parameter sources are detailed in the Methods and Table 1.

**Table 1:** Model inputs, base-case values, and probability distributions used in the economic model

Parameter	Base-case value	Distribution (PSA parameters)	Interpretation/rationale/sources
10-year CAD incidence (top 20% PRS)	12%	Beta (*α* = 120, *β* = 880)	Mean = 0.12; moderate uncertainty (effective *n* ~ 1000); reflects adult high-PRS incidence due to lack of pediatric data. Sources: UK Biobank & multi-ancestry PRS cohorts^3,24^
Relative risk reduction (CAD)	30%	Log-normal (RR)	Base-case assumes a 30% relative risk reduction (RRR), corresponding to a relative risk of 0.70; 95% interval for the relative risk approximately 0.60–0.80, consistent with effect sizes reported for lifestyle and statin-based primary prevention in adult populations. Sources: adult prevention trials^26,38^
10-year Stroke incidence (top 20% PRS)	12%	Beta (*α* = 120, *β* = 880)	Mean = 0.12; moderate uncertainty (effective *n* ~ 1000); assumed equal to CAD high-PRS incidence due to lack of pediatric stroke incidence data. Sources: cardiovascular epidemiology models^24,26,37^
Utility (age 10–40)	0.95	Normal (mean = 0.95, SD = 0.02)	High baseline utility in youth; modest variance. Sources: utility systematic reviews^39–41^
Utility (age 40–60)	0.92	Normal (mean = 0.92, SD = 0.03)	Gradual decline in utility with increasing age and comorbidity burden. Sources: utility systematic reviews ^39–41^
Utility (>60 years)	0.85	Normal (mean = 0.85, SD = 0.04)	Lower utility and wider uncertainty reflecting age-related disease burden. Sources: utility systematic reviews^39–41^
Acute CAD fatality rate	20%	Beta (α = 100, β = 400)	Mean = 0.20; effective n ~ 500; reflects uncertainty in epidemiologic case-fatality estimates. Sources: cardiovascular epidemiology models^26,37,39^
QALY loss per fatal CAD event	12.4 QALYs	Normal (mean = 12.4, SD = 1.2)	SD ~ 10% of mean; consistent with lifetime QALY loss estimates in CE models. Sources: U.S. cost-effectiveness models^26,37,39^
Acute stroke fatality rate	20%	Beta (*α* = 100, *β* = 400)	Mean = 0.20; effective n ~ 500. Assumed equal to the acute CAD case-fatality rate in the base-case due to lack of harmonized stroke-specific inputs; reflects uncertainty in epidemiologic case-fatality estimates. Sources: cardiovascular epidemiology models^26,37,39^
QALY loss per fatal stroke event	12.4 QALYs	Normal (mean = 12.4, SD = 1.2)	SD ~ 10% of the mean; consistent with lifetime QALY loss estimates used in cardiovascular cost-effectiveness models. Assumed equal to the CAD event QALY loss due to the lack of harmonized stroke-specific long-term utility estimates in this model. Sources: US cost-effectiveness models^26,37,39^
Genetic testing cost (per child)	$400	Gamma	Mean = $400; SD ~$80 (~20% CV); consistent with PRS commercial pricing. Sources: PRS cost-effectiveness analyses^17–19^
Statin + monitoring cost (annual)	$250	Gamma	Mean = $250; SD ~$50 (~20% CV); consistent with pediatric FH management costs. Sources: US medication and monitoring estimates
Lifetime direct CAD cost (per case)	$421,487	Gamma	Mean = $421,487; SD ~$105.000k (~25% CV); reflects US lifetime CAD treatment burden. Sources: US CAD cost studies^43–46^
Lifetime direct stroke cost (per case)	$421,487	Gamma	Mean = $421,487; SD ~$105.000k (~25% CV); Mean and uncertainty reflect major cardiovascular event cost estimates used in published economic evaluations. Assumed equal to CAD event costs due to the lack of harmonized stroke-specific long-term cost inputs in this model. Sources: US cardiovascular cost-effectiveness and cost-of-illness studies^43–46^
Discount rate	3%	Fixed	Standard parameter for economic evaluations. Source: Second Panel Guidelines^22^
Medical inflation	5%	Fixed	Reflects projected long-term healthcare expenditure growth. Sources: US health expenditure projections

For every parameter, the base-case value along with the probability distribution used in probabilistic sensitivity analyses (PSA) is provided. Within the interpretation column, the choice of parameter values, associated uncertainty, and literature references supporting these estimates are explained. Beta and gamma distributions were applied for proportions and costs, respectively. Normal distributions were used for utilities and QALY losses. Parameters such as discount rate and medical inflation were based on fixed values.

Projected life expectancy, quality-adjusted life years (QALYs), and cardiovascular risk across scenarios are reported in Table 2. The modeled values represent internally consistent model-based projections under illustrative scenario assumptions, derived from known gradients in cardiovascular risk.

**Table 2:** Projected life expectancy, QALYs, and cardiovascular risk across scenarios

Scenario	Life expectancy (years)	QALYs	MI risk (%)	Stroke risk (%)	Fatal MI (%)
Low PRS (baseline)	82	70.0	10	5	2
High PRS, no prevention	72	53.9	30	12	10
High PRS, with prevention	79	67.2	15	7	4

Life expectancy (estimated), total quality-adjusted life years (QALYs), and 10-year risks of myocardial infarction (MI), stroke, and fatal MI are presented for three groups: individuals with low PRS (baseline), those with high PRS without prevention, and those with high PRS who have undergone targeted preventive measures. Values represent model-based projections under illustrative scenario assumptions. These values are intended to illustrate relative differences across genetic risk strata and intervention scenarios and should not be interpreted as calibrated population-level projections. The base-case analysis applies a conservative 30% relative risk reduction derived from coronary artery disease data to both myocardial infarction and stroke; the larger reductions shown in the illustrative MI and stroke scenarios are presented for descriptive purposes only and were not used to parameterize the base-case model.

Low-PRS individuals were assigned a typical life expectancy of 82 years (~70 QALYs), whereas high-PRS individuals without prevention were modeled with shorter survival (72 years), lower utility (53.9 QALYs), and proportionally higher rates of MI, stroke, and fatal MI. Consequently, high-PRS individuals without prevention face a 10-year reduction in life expectancy, a 76% increase in MI risk, and a fivefold higher fatal MI risk. Preventive interventions were assumed to partially offset this excess risk, increasing life expectancy by 7 years and adding 13.3 QALYs.

### Program implementation costs

Implementation costs included genotyping, virtual counseling, and a lifestyle intervention program^17–19^ and statin-based preventive interventions for children identified as being at the highest risk (Table 3). A 30-year horizon was used to estimate long-term medication costs, with a discounted approach. We modeled outcomes over a 30-year horizon to balance long-term impact assessment with practical forecasting limits, while acknowledging that outcomes will extend over 30–70 years (Supplementary Table 1). The cost of genetic testing has decreased over the past few years and is expected to continue declining. The bulk of the cost is associated with the one-time expenditure of $4 million for 10,000 children. An additional cost for long-term statin therapy and monitoring was included for the cohort with a very high-risk score (top 2% PRS).

**Table 3:** Program implementation costs for PRS-guided CAD prevention

Component	Estimate
Screening + counseling + lifestyle intervention programs per Child	$400
Statin + monitoring annual cost (top 2%) per child	$250
Total genetic testing cost (10,000 children)	$4 million
30-Year medication cost (discounted at 3%)	$970,000
Total program cost (discounted)	$4.97 million

Genetic testing accounts for the majority of the cost, estimated at $4 million for 10,000 children. A smaller portion of the cost, $970,000, is attributed to long-term medications for the treated subgroup over 30 years.

### Direct healthcare and productivity savings

Key cost drivers include interventions, hospitalizations, medications, and long-term follow-up. Acute care accounts for early spikes, while chronic care costs accumulate over time, with an estimated lifetime cost of $421,487 per case (Supplementary Table 2). Compared with standard care, PRS-guided prevention is projected to prevent 72 CAD cases, generating $30.3 million in direct healthcare savings. Each CAD case yields an average gain of 13.3 QALYs. Preventing 72 cases yields 958 quality-adjusted life years (QALYs), valued at $100,000 each, totaling $95.8 million in economic benefits^20^. Productivity gains are estimated at $200,000 per CAD case, and preventing 72 cases provides an additional $14.4 million in productivity savings (Table 4).

**Table 4:** Estimated lifetime clinical and economic impact of PRS-Guided CAD prevention per 10,000 children

Metric	Estimate
Lifetime direct cost of CAD (per case)	$421,487
Total CAD cases prevented	72 cases
Direct healthcare savings	$30.3 million
QALYs gained per case	13.3 years
Total QALYs gained	958 QALYs
Monetary value per QALY	$100,000
Total value of QALYs gained	$95.8 million
Productivity savings per case	$200,000
Total productivity gains	$14.4 million
Screening cost savings from PRS stratification	$20 million
Indirect health co-benefits (15% multiplier)	$24.1 million
Total societal benefit	$184.6 million
Program implementation cost (discounted)	$4.97 million
Return on Investment (ROI)	3614%
Adjusted ROI	6515%

Total societal benefit: $184.6 million and program cost of $4.97 M, yielding an ROI of approximately 3614%. The adjusted ROI of 6515% reflects the joint application of a 5% annual inflation rate and a 3% annual discount rate over 30 years.

### Screening cost savings from PRS stratification

Modeling cardiovascular screening strategies in a cohort of 10,000 adults revealed that uniform annual screening for all individuals would cost approximately $75 million (undiscounted). In contrast, a PRS-guided stratified approach—offering intensive screening for 2000 high-risk individuals starting at age 40 and less frequent screening for others starting at age 50—reduces total costs to $55 million, resulting in $20 million in savings (Supplementary Table 3). This risk-based strategy enhances economic efficiency by aligning screening intensity with genetic risk.

### Indirect health co-benefits

Lifestyle and preventive interventions targeting CAD may offer broader health benefits by reducing the incidence of comorbid conditions such as type 2 diabetes, certain cancers, and depression. Based on our estimates from multifactorial public health models, including those evaluating diet, physical activity, and comprehensive lifestyle programs, a conservative 15% co-benefit multiplier was applied to account for these indirect health gains. Incorporating this multiplier yields $ 24.1 million in aggregate indirect benefits (Table 4).

### Cost-effectiveness and break-even analysis

Preventing just 2–3 cases offsets program costs, supporting feasibility even in resource-constrained settings. The incremental cost-effectiveness ratios (ICER), representing the additional cost required to gain one QALY, were $5188. This ICER is well below conventional willingness-to-pay thresholds ($50,000–$100,000 per QALY), indicating strong cost-effectiveness.

### Adjusted ROI accounting for inflation and discounting

We estimated an unadjusted return on investment (ROI) of 3614%, based on projected societal benefits of $184.6 million in today’s dollars and a program cost of $4.97 million. To account jointly for projected healthcare cost inflation (5% annually) and discounting of future costs and benefits (3% annually), we estimated an inflation- and discount-adjusted net present value. Under these assumptions, the corresponding adjusted return on investment was 6515%, reflecting an inflation- and discount-adjusted net present value of $328.8 million over 30 years.

Although we report the unadjusted ROI in the main text for comparability with existing studies, the adjusted figure more accurately captures the long-term financial value.

### Probabilistic sensitivity analysis

**Fig. 1:**
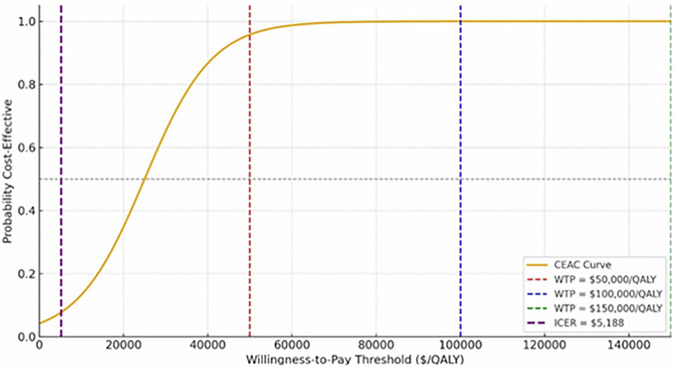
Cost-effectiveness acceptability curve across WTP thresholds. The cost-effectiveness acceptability curve (CEAC) illustrates the probability that the PRS-guided CAD prevention program is cost-effective across a range of willingness to pay (WTP) thresholds. The program achieves a probability of cost-effectiveness exceeding 95% at a WTP of approximately $50,000 per quality-adjusted life year (QALY) and reaches near-certainty above $60,000 per QALY. The vertical purple line marks the calculated ICER of $5188 per QALY, demonstrating that the intervention is highly cost-effective, even at conservative WTP thresholds widely used in health policy decision-making.

Cost-effectiveness acceptability curves (CEACs) were plotted across a range of willingness-to-pay thresholds (Fig. 1). Relative to standard care, PRS-guided prevention yielded an ICER of $5188 per QALY gained and demonstrated robust performance across key outcomes. The probability of cost-effectiveness at a $50,000/QALY willingness-to-pay threshold exceeded 99% (Table 5). Model parameters used in the PSA are presented in Supplementary Table 4.

**Table 5:** Probabilistic sensitivity analysis (PSA) results based on 1000 Monte Carlo simulations

Outcome	Mean	95% Confidence interval
ROI	3647%	2781–4672%
CAD cases prevented	72 cases	58–86 cases
QALYs gained	959 QALYs	756–1156 QALYs
Probability is cost-effective at $50,000/QALY	>99%	–

Mean values and 95% confidence intervals (CI) are shown for return on investment (ROI), number of coronary artery disease (CAD) cases prevented, total quality-adjusted life years (QALYs) gained, and the probability of cost-effectiveness at a willingness-to-pay threshold of $50,000 per QALY.

Estimates of ROI and ICER were adjusted to reflect local healthcare costs, value per QALY, and national spending patterns in high-income countries. Despite regional differences, the PRS-guided prevention program remained highly cost-effective and demonstrated a strong return on investment (ROI) across all countries assessed (Table 6).

**Table 6:** Country-specific ICER and ROI estimates adjusted for local economic parameters

Country	ICER (USD per QALY)	ROI (%)
USA	$5188	3614
UK	$3891	3119
Germany	$4150	3382
Canada	$4410	3396
Australia	$4669	3202
France	$4410	3177

Incremental cost-effectiveness ratios (ICERs) in USD per quality-adjusted life year (QALY) gained, and corresponding ROI percentages are presented for the USA, UK, Germany, Canada, Australia, and France, adjusted for local healthcare costs and spending patterns.

## Discussion

Our findings indicate that PRS-guided prevention in childhood offers meaningful long-term clinical benefit and is highly cost-effective in high-income settings. By identifying high-risk children early and targeting preventive interventions accordingly, this approach has the potential to shift cardiovascular prevention upstream and substantially reduce future disease burden. Our model projects a return of $36 for every $1 invested—exceeding or approaching the economic returns of widely adopted programs such as childhood vaccinations (~$10 per $1)^21–23^.

PRS-guided prevention remains highly cost-effective, and the one-time cost of genotyping becomes even more favorable when applied across multiple disease areas. To ensure a conservative estimate, we assumed a 10-year CAD incidence of 12% among individuals in the top quintile of PRS^24^. However, recent multi-ancestry studies report incidence rates approaching 20%^25^, indicating that our model may underestimate the potential clinical and economic impact. Moreover, applying this model to populations with higher baseline CAD prevalence and severe clinical manifestations than those observed in the UK Biobank would likely result in a greater number of cases averted and further enhance the projected cost-effectiveness.

Notably, our projections are based on adult data where preventive interventions typically begin at age 40 or later. In contrast, initiating PRS-guided prevention in childhood is likely to yield greater benefit. Atherosclerosis begins in early life, and earlier intervention provides a longer window for modifying risk, stronger behavioral imprinting, and greater biological responsiveness during development^26,27^. As such, the modeled 30% risk reduction is likely conservative and may underestimate the true impact of early PRS-guided prevention.

This rationale is supported by longitudinal studies such as the Bogalusa Heart Study and the Young Finns Study, which show that childhood cardiovascular risk factors strongly predict adult outcomes^1,2^. Mendelian randomization studies further demonstrate that early reduction in LDL-C confers durable protection against cardiovascular disease^28^. While familial hypercholesterolemia (FH) explains only a fraction of early CAD, polygenic risk accounts for approximately 20% of early-onset myocardial infarctions^9,24,26^. The total CAD heritability is estimated to be 40–60%, primarily due to common polygenic variants^3,29,30^.

High PRS has been associated with early vascular changes, supporting its utility in primordial prevention^6–8^. When used in conjunction with routine lipid screening at ages 9–11, PRS enhances clinical sensitivity and risk stratification beyond LDL-C alone^9,20,24^. Early identification enhances timely lifestyle intervention and statin therapy, which may alter the long-term trajectory of cardiovascular risk. Pediatric statin trials, such as the COPE study, have demonstrated vascular benefits and a lasting “legacy effect” from early treatment^27,31^.

Our model has limitations common to early economic evaluations. To avoid overstating the benefit, we applied conservative assumptions, despite evidence suggesting potentially greater benefit with earlier intervention. While the original schematic allowed for the recurrent events conceptually, the base-case implementation did not include separate recurrence probabilities, costs, or utilities. This represents a limitation; however, explicitly modeling recurrent events would likely increase the projected clinical benefits and cost-effectiveness. Our model has not yet been externally validated against real-world pediatric PRS-guided prevention data; future prospective studies will be critical for testing and refining these projections. In the absence of external validation, probabilistic sensitivity analyses provide a structured assessment of parameter uncertainty, and these analyses showed robust results. We did not incorporate disutility related to treatment burden or psychological effects of risk labeling, which may lead to overstating the benefit. Although concerns about treatment burden and the psychological consequences of labeling a child “very high risk” must be acknowledged, evidence from adult preventive genomics shows that structured disclosure of monogenic and polygenic CAD risk generally increases preventive engagement and does not result in sustained psychological harm^30,32,33^. Long-term adherence remains uncertain. Pediatric FH programs report ~75–80% adherence at 10 years^34^, but adherence across chronic pediatric conditions is typically lower. Our model reflects an optimistic adherence scenario; lower real-world adherence would attenuate absolute benefit and cost-effectiveness. The model does not account for non-CAD mortality or morbidity prior to age 40, which is low in high-income settings but may slightly overestimate benefit. Rare monogenic forms of CAD fell beyond the model’s scope. Although tailored to high-income settings, the framework is adaptable, and future prospective validation in diverse pediatric populations is warranted.

Despite its promise, PRS-guided prevention faces significant challenges in implementation. Our bibliometric analysis revealed a marked scarcity of pediatric cardiometabolic PRS studies focusing on CAD, with most conducted in high-income, European ancestry populations^12–15^. This limits generalizability and may reduce predictive accuracy in underrepresented groups. Without correction, Eurocentric models risk widening existing health disparities. Addressing this gap requires ancestry-informed development and validation of polygenic risk scores (PRS) in diverse pediatric cohorts.

Ethical and operational considerations are equally important. Early disclosure of genomic risk may raise concerns about anxiety, stigma, and insurance discrimination. These concerns can be mitigated through transparent communication, informed parental assent, and alignment with existing ethical guidelines from bodies such as the American College of Medical Genetics and Genomics (ACMG) and American Academy of Pediatrics (AAP)^35^. Long-term adherence to preventive strategies remains a significant challenge, particularly in low-resource settings.

Operationally, PRS could be integrated into newborn screening programs or routine pediatric visits, with longitudinal tracking facilitated through electronic health records (EHRs). However, the integration of genetic risk into pediatric EHRs necessitates stringent data privacy protections, long-term storage protocols, and governance frameworks to safeguard sensitive genomic information. Embedding PRS within pediatric care workflows—guided by established frameworks, such as the American Heart Association’s Life’s Essential 8 offers a shift from reactive treatment to proactive, upstream prevention. Realizing this transition requires significant investment in provider training, genomic literacy, and system-wide readiness to operationalize PRS within the framework of preventive care. In high-burden settings such as the United States, where cardiovascular expenditures are projected to exceed $1.8 trillion by 2050^36^; this upstream, genomically guided strategy may offer substantial long-term cost savings and public health benefits.

In summary, PRS offers a promising tool for early CAD prevention, with the potential to improve long-term cardiovascular outcomes and redefine the landscape of pediatric preventive cardiology. Pilot implementation in high-risk pediatric populations or national early screening initiatives is warranted to evaluate clinical feasibility, cost-effectiveness, and scalability.

## Methods

### Bibliometric search strategy

We conducted a bibliometric and narrative review to map and analyze the global research output on PRS in pediatric populations related to cardiometabolic disease and CAD. We used keywords pertaining to PRS and pediatric populations, in combination with CAD and cardiometabolic terms. We employed the following search strategy: TS = (“child” OR “infant” OR “adolescent” OR “pediatrics” OR “teen*“ OR “youth” OR “juvenile”)) AND (TS = (“polygenic risk score” OR “genetic risk score” OR “polygenic scores”)) AND (TS = (“cardiometabolic” OR “coronary artery disease” OR “blood pressure” OR “hypertension” OR “lipids” OR “obesity” OR “BMI” OR “diabetes” OR “insulin resistance”)). The Web of Science (WoS) core collection was selected as the primary data source for its comprehensive indexing of high-impact journals and structured bibliometric metadata. A sensitivity analysis using the same search strategy in PubMed revealed a variation of less than 5% in the number of articles. We included studies published between January 1, 1999, and December 31, 2024, limited to English-language original research articles. To capture very recent research in progress, we also included meeting abstracts.

### Model structure

Building on the evidence gap identified in our bibliometric review, we developed a theoretical decision-analytic model to project the potential long-term clinical and economic effects of pediatric PRS-guided prevention. Because longitudinal pediatric CAD-PRS data do not yet exist, adult-derived incidence and treatment-effect estimates provide the only available evidence base to inform projections into adulthood.

We developed a theoretical Markov economic model to estimate the long-term clinical and financial outcomes of implementing PRS-guided prevention strategies for CAD in children. The model adopted a societal perspective and incorporated epidemiologic stratification, cost-benefit analysis, and Quality-adjusted life years (QALYs)^9,21,22^, adhering to CHEERS 2022 guidelines^24^.

The model evaluates PRS-guided prevention versus standard care. In the standard-care arm, children receive guideline-concordant pediatric preventive care (routine visits, family history assessment, lifestyle counseling, and lipid screening), with pharmacologic therapy initiated only when clinically indicated (e.g., familial hypercholesterolemia or markedly elevated LDL-cholesterol). Standard care does not include PRS testing, PRS-based risk stratification, or PRS-guided preventive interventions. A cohort of 10,000 children enters the model at age 10 and is followed annually to age 80 or death. Screening and intervention begin at age 10, but CAD outcomes are simulated starting at age 40, reflecting a 30-year intervention period.

The model includes four mutually exclusive states: (1) alive without CAD or stroke, (2) post-nonfatal cardiovascular event (MI or stroke), (3) death from cardiovascular causes (fatal MI or stroke), and (4) death from non-cardiovascular causes. Each year, individuals may remain event-free, experience nonfatal MI or stroke, experience fatal MI or stroke, or die from non-CAD causes. Death states are absorbing.

**Fig. 2:**
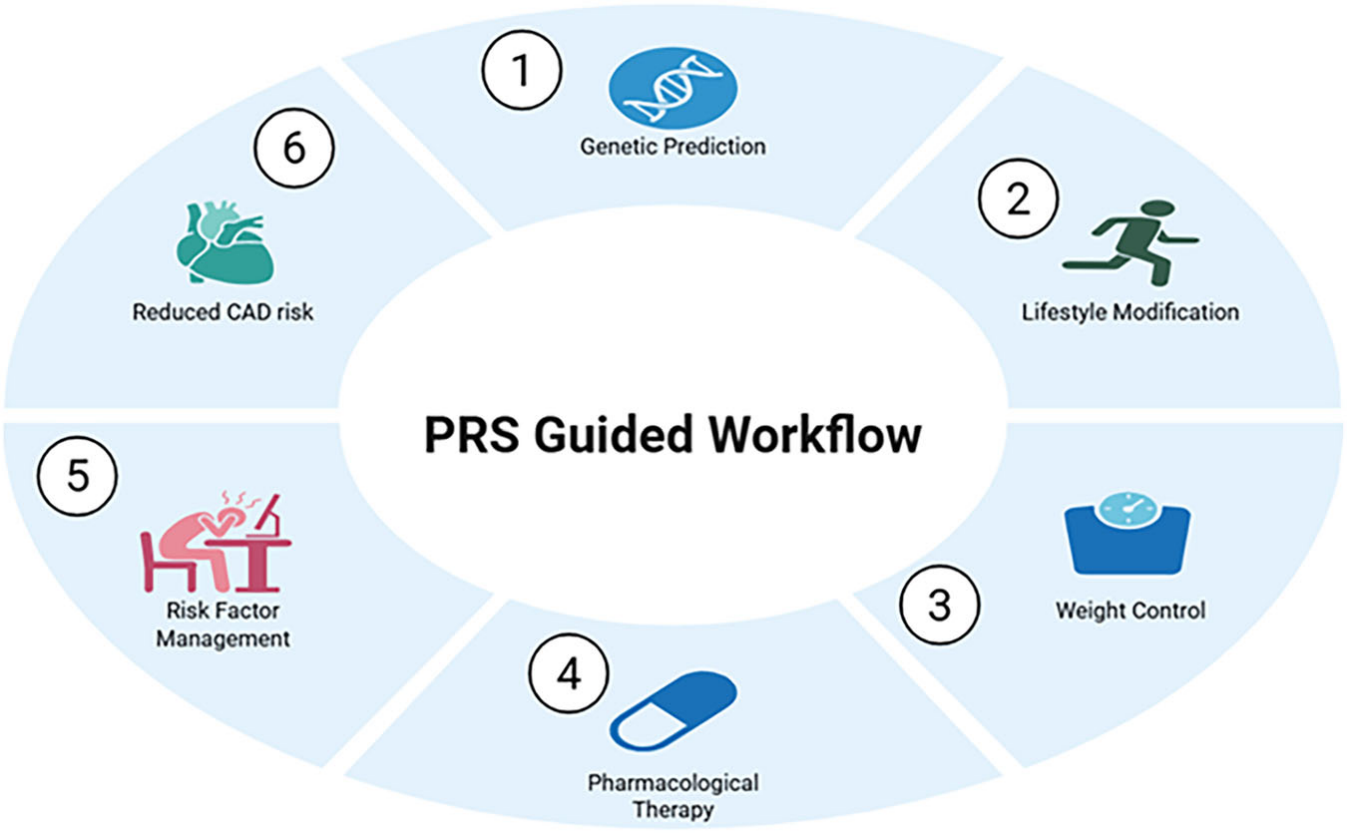
PRS guided workflow for early coronary artery disease prevention in children. This conceptual framework outlines the key steps of PRS-guided workflow implementation. Genetic risk stratification informs lifestyle modification, weight control, pharmacological therapy, and risk factor management, resulting in reduced CAD risk.

All children begin in a healthy state. In the PRS-guided arm, children in the top 20% PRS receive intensive lifestyle intervention, and those in the top 2% additionally receive statin therapy (Fig. 2). Transition probabilities for MI, stroke, and cardiovascular mortality are derived from published cohorts and life tables, with PRS modifying cardiovascular event risk from age 40 onward.

### Societal benefit and global applicability

Cost inputs were derived from published literature and adjusted to 2024 US dollars using the gross domestic product (GDP) deflator, with 2019 as the base year. Routine pediatric preventive care was assumed to be delivered similarly in both arms and therefore does not differ between strategies. Consequently, routine care costs were assumed to cancel out in the incremental analysis and were not included explicitly. Only the incremental costs related to PRS testing, PRS disclosure and counseling, intensified prevention in high-risk strata, and downstream differences in cardiovascular event costs were modeled. Costs included one-time genetic testing and counseling for all children, as well as ongoing medication and monitoring expenses for those who received treatment, all of which were discounted at a rate of 3% annually over a 30-year period. For broader applicability, inputs were adjusted for other high-income countries (e.g., Canada, Australia, and EU nations), with cost estimates scaled to GDP per capita and QALY valuations aligned with local Health Technology Assessment (HTA) thresholds. All costs were updated to 2024 values. Because currently available CAD polygenic risk scores were developed primarily in European-ancestry populations, the base-case analysis reflects performance in similar populations. Predictive accuracy may differ across underrepresented ancestries, underscoring the need for local validation before clinical implementation.

### Model parameters

All base-case parameters were prespecified and derived from published epidemiologic studies, meta-analyses, clinical trials, and cost-effectiveness guidelines (Table 1), following CHEERS 2022 standards. For the assessment of CAD Incidence and PRS gradient, we utilized a 10-year CAD incidence of 12% for individuals in the top 20% of the PRS distribution, derived from UK Biobank and multi-ancestry genomic cohorts and used as an adult benchmark^3,24^. Individuals in the top 20% of the PRS distribution have a threefold higher risk of CAD compared to the population average^25^. Based on adult intervention studies reporting relative risk reductions ranging from approximately 20% to 40%, we conservatively applied a uniform 30% relative risk reduction^12,26,37,38^. We applied the relative risk reduction of 30% to clinical atherosclerotic cardiovascular events, including both myocardial infarction and stroke. All base-case analyses use this uniform 30% relative risk reduction for both myocardial infarction and stroke; higher relative reductions shown in Table 2 reflect illustrative scenario assumptions only and were not used to parameterize the base-case model. Age-specific utility weights were obtained from cardiovascular systematic reviews and established decision-analytic models^39–41^ and applied uniformly across scenarios. We modeled fatality and QALY loss according to a 20% acute CAD fatality rate and a loss of 12.4 QALYs per fatal event based on established cardiovascular modeling studies^26,37,39^. Stroke case-fatality, utility loss, and event costs were conservatively assumed to be equal to major coronary event parameters owing to the lack of harmonized long-term stroke-specific inputs. Cost inputs included the aggregate of testing, intervention, and healthcare costs ascertained from international economic analyses (2016–2024)^17–19^, inflation-adjusted to 2024 USD, and scaled for high-income health-system contexts. A 3% annual discount rate and 5% medical inflation rate were applied per Second Panel recommendations^22^. All assumptions were intentionally conservative to avoid inflating the projected benefits of PRS-guided prevention.

### Probabilistic sensitivity analysis

A probabilistic sensitivity analysis (PSA) was performed using 1000 Monte Carlo simulations. Probability parameters (e.g., disease incidence and adherence) were modeled using beta distributions, cost variables using gamma distributions, and QALYs using normal distributions^42^. Output metrics included return on investment (ROI), incremental cost-effectiveness ratios (ICERs), and cost-effectiveness acceptability curves (CEACs).

All PSA distributions were centered on the base-case values and parameterized to reflect moderate uncertainty around published estimates, with full distribution parameters (e.g., alpha and beta for beta distributions; shape and scale for gamma distributions; mean and SD for normal distributions) reported in Table 1.

## Supplementary information

supplementary_info

## Data Availability

The data that support the findings of this study are not openly available due to reasons of sensitivity and are available from thecorresponding author upon reasonable request.

## Code availability

The code used in this study is not publicly available but may be provided to qualified researchers upon reasonable request to the first or corresponding author.
