# Supplementary material for: Economic modeling of polygenic risk prediction of coronary artery disease in childhood
Source: NPJ Cardiovasc Health. 2026 Mar 23;3:13. doi: 10.1038/s44325-026-00110-z (PMC13009156; doi:10.1038/s44325-026-00110-z)
Supplement: Supplementary file 1 — supplementary_info [file 44325_2026_110_MOESM1_ESM.pdf]

Supplemental Material

Economic Modeling of Polygenic Risk Prediction of Coronary Artery Disease in Childhood

Fouad Bitar[1,2], Rana Zareef[3], Hussain Ismaeel[4], Roukoz Abou-Karam[1,2], Mariam Arabi[3], Ziad Bulbul[3], Fadi F. Bitar[3#*], Akl C. Fahed[1,2#*]

[1] Cardiovascular Disease Initiative, Broad Institute of MIT and Harvard, Cambridge, MA, USA

[2] Division of Cardiology, Massachusetts General Hospital, Harvard Medical School, Boston, MA, USA

[3] Children's Heart Center, Department of Pediatrics and Adolescent Medicine, American University of Beirut-Medical Center, Lebanon

[4] Division of Cardiology, Aman Hospital, Doha, Qatar

# Co-senior authors

*** Corresponding Authors:**

Akl C. Fahed, email: afahed@mgh.harvard.edu.

Fadi Bitar, email : fbitar@aub.edu.lb

Supplementary Figure 1. Annual publication output on polygenic risk scores for cardiometabolic traits in children

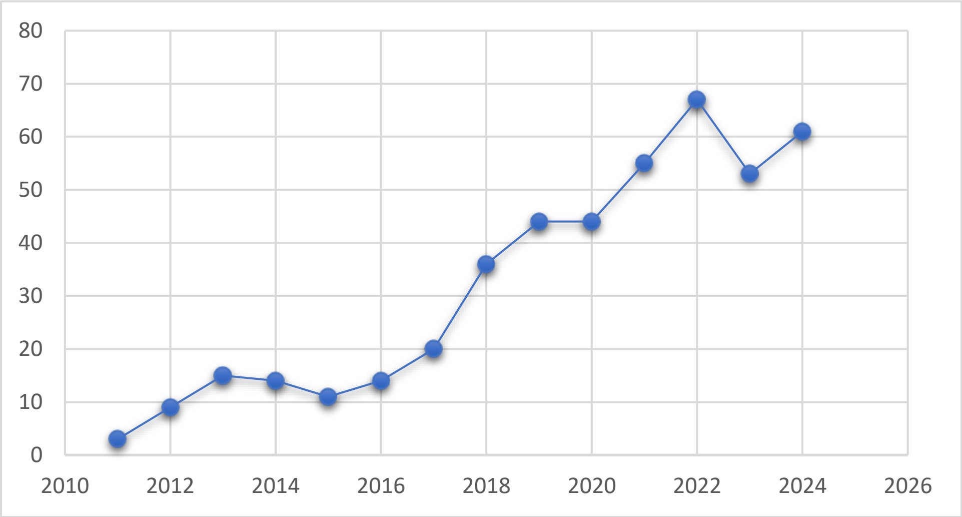

Each blue circle represents the annual publication across the associated year.

Supplementary Table 1. Key Model Assumptions and Intervention Parameters for PRS-Guided Prevention in a Pediatric Cohort

Parameter	Value
Population	10,000 children
High-risk (top 20%)	2,000 children
Very High-risk (top 2%)	200 children
Screening Age	10 years
Risk Stratification	PRS-defined low, moderate, and high risk
Time Horizon	30–70 years
Intervention	Lifestyle counselling and targeted modification, diet, pharmacologic therapy, and monitoring.
Comparator	Standard care
Discount Rate	3% annually
Medical Inflation Rate	5% annually

Supplementary Table 2. Cost of Cardiovascular Events

Component	USA
Acute Care Cost (per episode)	$20900
Chronic Care Cost (annual)	$21086
Lifetime Chronic Cost	$316290
Total Lifetime Cost (Int$ 2019)	$337190
Inflation Adjustment (2019→2024)	1.25
Total Lifetime Cost (2024 Currency)	$421487.5

All cost estimates were adjusted to 2024 U.S. dollars using the GDP deflator, with 2019 as the base year, in accordance with recommended health economic practices.

Supplementary Table 3. Screening Savings by PRS-Guided Stratification

Variable	Value
Cost per person – Uniform screening	$7,500
Cost per person – High PRS screening	$20,000
Cost per person – Low-intermediate PRS screening	$1,875
Total cost – Uniform screening	$75 million
Total cost – PRS-guided screening	$55 million
Total savings with PRS-guided strategy	$20 million

Supplementary Table 4. Model Input Parameters and Ranges for Probabilistic Sensitivity Analysis (PSA) in PRS-Guided CAD Prevention

Parameter	Base Value	Range (for PSA)
Screening Age	10 years	Fixed
High PRS prevalence (Top 20%)	20%	15–25%
CAD Risk Reduction with Prevention	30%	20–40%
Cost of PRS Screening per case	$400	$200–$400
Annual Statin + Monitoring Cost	$250	$200–$300
Acute Care Cost per CAD Event	$20,900	$15,000–$30,000
Chronic Care Annual Cost	$21,086	$15,000–25,000
Discount Rate	3%	2%–5%
Inflation Rate	5%	3%–6%
